# Use of partial least squares regression to impute SNP genotypes in Italian Cattle breeds

**DOI:** 10.1186/1297-9686-45-15

**Published:** 2013-06-05

**Authors:** Corrado Dimauro, Massimo Cellesi, Giustino Gaspa, Paolo Ajmone-Marsan, Roberto Steri, Gabriele Marras, Nicolò PP Macciotta

**Affiliations:** 1 Dipartimento di Agraria, Sezione Scienze Zootecniche, Università di Sassari, Sassari, 07100, Italy; 2 Istituto di Zootecnica, Università Cattolica del Sacro Cuore, Piacenza, 29100, Italy; 3 Consiglio per la Ricerca e la Sperimentazione in Agricoltura, via Salaria 31, Monterotondo, 00015, Italy

## Abstract

**Background:**

The objective of the present study was to test the ability of the partial least squares regression technique to impute genotypes from low density single nucleotide polymorphisms (SNP) panels i.e. 3K or 7K to a high density panel with 50K SNP. No pedigree information was used.

**Methods:**

Data consisted of 2093 Holstein, 749 Brown Swiss and 479 Simmental bulls genotyped with the Illumina 50K Beadchip. First, a single-breed approach was applied by using only data from Holstein animals. Then, to enlarge the training population, data from the three breeds were combined and a multi-breed analysis was performed. Accuracies of genotypes imputed using the partial least squares regression method were compared with those obtained by using the Beagle software. The impact of genotype imputation on breeding value prediction was evaluated for milk yield, fat content and protein content.

**Results:**

In the single-breed approach, the accuracy of imputation using partial least squares regression was around 90 and 94% for the 3K and 7K platforms, respectively; corresponding accuracies obtained with Beagle were around 85% and 90%. Moreover, computing time required by the partial least squares regression method was on average around 10 times lower than computing time required by Beagle. Using the partial least squares regression method in the multi-breed resulted in lower imputation accuracies than using single-breed data. The impact of the SNP-genotype imputation on the accuracy of direct genomic breeding values was small. The correlation between estimates of genetic merit obtained by using imputed versus actual genotypes was around 0.96 for the 7K chip.

**Conclusions:**

Results of the present work suggested that the partial least squares regression imputation method could be useful to impute SNP genotypes when pedigree information is not available.

## Background

In genomic selection programs, the breeding value (GEBV) of an individual is assessed by combining both genomic and traditional pedigree-based predictions. High-density marker platforms (HDP) of different SNP (single nucleotide polymorphism) densities (50K and 777K) are currently used to genotype bulls under selection [1] and elite cows and to test for marker-phenotype associations [2,3].

Genotyping costs are among the major constraints for large-scale implementation of genomic selection in many breeds. However, the commercial availability of low density SNP panels (LDP), such as the Illumina Bovine3K Genotyping BeadChip or the Illumina BovineLD BeadChip, which contains around 7K markers [4], has offered new opportunities to increase the number of animals involved in selection programs. Genotypes obtained from an LDP must be imputed to the 50K platform by using suitable algorithms. Genotype imputation can also be useful when combining data sets that were generated using different SNP chips [5].

Genotype imputation refers to *in silico* reconstruction of missing genotypes. Several techniques have been proposed to routinely impute SNP genotypes. The following three steps are common to all procedures: (1) a training population (TP) genotyped with an HDP is created; (2) a prediction population (PP) is generated by using an LDP; and (3) a suitable algorithm is used to impute missing SNPs in the PP.

On the basis of the information considered to infer missing marker genotypes, imputation methods can be classified into three groups. The first relies on linkage and family information [6,7], the second uses linkage disequilibrium based on population information [8,9], and the third combines the two former sources of information [10,11]. Several factors affect imputation accuracy. In particular, imputation accuracy strongly depends on the number of individuals in the training population and on the marker density of the LDP [10,12-14].

The impact of imputed genotypes on GEBV accuracies has been investigated. Results are sometimes discordant or expressed in different ways. For example, Chen et al. [15] compared GEBV values obtained with actual and imputed data. Two computer programs, Findhap [11] and Beagle [9], were used to impute SNP genotypes from a 3K panel to a 50K panel. The loss of reliability in GEBV prediction by using imputed data was around 6.5% and 2.6% with Findhap and Beagle, respectively. Recently, Segelke et al. [16] reported a reduction in reliability of genomic predictions, averaged over 12 traits, ranging from 5.3% to 1% for the 3K and 7K chips, respectively. Moser et al. [17] proposed the use of an LDP that included the highest ranked SNPs for a trait under study. However, the gain in accuracy of GEBV obtained with the highest ranked SNP was only slightly higher (5–6%) than the accuracy obtained with an equal number of evenly spaced markers. Nevertheless, with this strategy, considering that a specific pool of markers is required for each trait, the use of evenly spaced SNP seems to be preferable over choosing a specific SNP set for each trait.

Several imputation algorithms have been proposed and implemented in freely available software such as Beagle [9], DAGPHASE [10] and Findhap [11]. Chen et al. [15] found Beagle to be the most accurate but at the expense of longer computation time.

A method that uses the Partial Least Squares Regression (PLSR) technique to impute SNP genotypes was proposed recently [18]. It was tested on a simulated genome consisting of 6000 SNPs equally distributed on six chromosomes and a data set of 5865 individuals (TP = 4665 and PP = 1200). The PLSR method yielded accuracies in marker imputation ranging from 0.99 to 0.86 when 10% or 90% genotypes were imputed, respectively. In the latter case, the accuracy of direct genomic values (DGV) dropped from 0.77 to 0.74. Furthermore, Dimauro et al. [18] highlighted that, with a fixed percentage (50%) of SNPs to be predicted, imputation accuracies slowly decreased from 98% with TP = 5000, to 87% with TP = 1000 and to 69% with TP = 600. PLSR requires only genotype data, and other data, such as pedigree relationships, is not needed. Therefore, this approach could be useful when the population structure is not known.

The aim of the present work was to test the PLSR imputation method on real data. In particular, a scenario with a 50K genotyped TP and a PP genotyped using either the 3K or 7K panel was simulated. Moreover, the ability of the PLSR method to predict SNP genotypes for different bovine breeds and in a multi-breed approach was tested.

## Methods

### Data

Data consisted of SNP genotypes belonging to 2179 Italian Holstein bulls genotyped with the Illumina 50K Beadchip (single-breed dataset). Only markers located on the 29 autosomes were considered. Monomorphic SNPs and SNPs with more than 2.5% missing values were discarded. No editing for minor allele frequency (MAF) was applied. A total of 43 427 SNPs were retained and any missing genotypes for these SNPs were replaced by the most frequent genotype at that locus. Data on a total of 86 bulls were discarded, of which 48 were replicates or had inconsistent Mendelian inheritance information, and 38 had a low overall call rate (lower than 95%).

To study the performance in a multi-breed sample, 749 Brown Swiss and 470 Simmental bulls were also available. For the multi-breed data set, data from the three breeds were edited together to obtain the same SNPs in all data sets. At the end of the editing procedure, 30 055 markers were retained.

Genotypes were coded according to the number of copies of a given SNP allele they carried, i.e. 0 (homozygous for allele B), 1 (heterozygous) or 2 (homozygous for allele A). The phenotypes available for all animals were polygenic estimated breeding values for milk yield, protein and fat content. Animals were ranked according to their age: the oldest were designed as TP with all genotypes considered known, whereas the youngest represented the PP. For both the single and multi-breed approach, SNPs belonging to 3K and 7K LDP were identified in the PP animals and all other genotypes were masked, thus mimicking the two Illumina LDP.

### The partial least squares regression imputation method

PLSR is a multivariate statistical covariance-based technique that is able to predict a response matrix **Y**_(*n × p*)_ from a predictor matrix **X**_(*n × m*)_ and to describe the common structure of the two matrices [18]. In both **X** and **Y**, *n* represents the number of animals involved, *m* is the number of SNPs in the LDP and *p* is the number of SNPs to be imputed. PLSR allows for the identification of underlying variables (known as latent factors) which are linear combinations of the explanatory variables **X**, that best model **Y**. Dimauro et al. [18] demonstrated that the accuracy of PLSR prediction increases with the number of latent factors approaching the number of SNPs to be predicted (the columns of **Y**). The maximum number of latent factors depends on the size of **X**, which has a lower number of columns than **Y**. For this reason, in each run, the number of extracted latent factors was fixed to be equal to the number of predictors (the number of columns of **X**). PLSR is a multivariate statistical technique particularly useful in genomic studies in which a great number of variables are involved. It can overcome the strong collinearity between SNP variables in **X** or **Y** and, at the same time, maximize correlations between **Y** and **X** variables [18,19]. A more detailed description of the PLSR imputation method can be found in Dimauro et al. [18].

In the present work, each chromosome was processed independently and data were analyzed by using the PLS procedure of SAS® software (SAS® institute Inc., Cary, NC). Datasets were organized in a multivariate manner, having SNPs as columns and animals as rows. The 50K SNPs were divided into SNPs that have to be imputed (**Y**) and SNPs used as predictors (**X**). In particular, **X** contained only SNPs belonging to the 3K or 7K LDP. For animals in the PP, genotypes in **Y** were masked and constituted the SNPs to be predicted.

### Genotype imputation from 3K (7K) LDP to the 50K SNP panel

The comparison of imputation performances from different publications is difficult due to the many differences between studies. TP size and number of markers in LDP heavily affect the accuracy of prediction. Moreover, the relationships between training and validation animals have an impact on imputation accuracies [20]. So, before applying the PLSR imputation method to our data, the method was tested on external data provided by Daetwyler et al. [6] who exploited the ChromoPhase program [6] to impute missing genotypes from low to high density SNP platforms. The data consisted of 1183 Holstein bulls genotyped with the Illumina 50K chip. Only the 2529 markers on chromosome 1 were available. A PP genotyped with the 3K chip (182 SNP) was simulated by masking the markers not present on the 3K chip. In particular, the PP was divided into non-founders (112 individuals that have at least one genotyped parent) and founders (212 animals that do not have a genotyped parent) and imputation accuracies were evaluated for both categories of animals. The PLSR method and Beagle [9] software were used to impute SNP genotypes in the PP and results were compared with accuracies obtained by Daetwyler et al. [6]. Population structure or pedigree was not used with either method.

In our experimental data, PLSR was first applied to the Holstein breed. Animals were ranked by age and divided in TP = 1993 (the older bulls) and PP = 100 (the younger) and both 3K and 7K scenarios were investigated. The Beagle software was applied to the same data. No pedigree information was used for either PLSR or Beagle.

On simulated data, Dimauro et al. [18] demonstrated that, for each chromosome, the PLSR imputation accuracy improved as the number of variables contained in **X** increased. The reason is that when many variables have to be predicted (the columns of the **Y** matrix), the number of extracted latent factors should be large. The maximum number of possible latent factors is, however, less or equal to the number of variables in **X**. So, for chromosomes with a relatively low number of markers in **X**, a lower PLSR predictive ability is expected. This hypothesis can be easily tested by comparing the imputation accuracies obtained in the 3K and 7K scenarios. Moreover, a PLSR run using an **X** matrix obtained by combining SNPs belonging to chromosomes 26, 27 and 28, was carried out to test for possible improvement in genotype imputation accuracy when **X** is artificially enlarged.

### Genotype imputation from 3K LDP to the 50K SNP panel for different breeds

The availability of a sufficiently large TP is a crucial factor for genotype imputation. Therefore, it is interesting to investigate if a multi-breed TP could enhance the accuracy of genotype predictions. Some authors [21,22] reported a slight advantage of using a multi-breed TP to evaluate the genetic merit of animals under selection. However, Hayes et al. [23] showed that, in sheep breeds, accuracy of imputation in single-breed analyses was higher than accuracy of imputation in a multi-breed analysis. To test the PLSR method in a multi-breed context, three groups of animals, one for each breed, were selected. Each group contained 479 bulls (the size of the Simmental population) and was split into a TP of 379 and a PP of 100 individuals. The imputation was first performed separately for each single breed and then by combining the three groups, thus obtaining a multi-breed dataset with TP = 1137 and PP = 300 bulls.

### Evaluation of imputation accuracy

The ability of PLSR to impute SNP genotypes was quantified by considering the allele imputation error rate. This index represents the number of falsely imputed alleles divided by the total number of imputed alleles [14]. In practice, considering the real and the imputed genotypes, 0 error was counted if both genotypes were identical, 1 if the real genotype was homozygous and the imputed genotype heterozygous (or vice versa) and 2 if the real and imputed genotypes were both homozygous but different. The imputation accuracy (R), for each SNP, was equal to 1 minus allele error rate. The allele error rate and the related imputation accuracy were averaged both by chromosome and across all chromosomes.

The effect of SNP imputation on accuracy of DGV was also evaluated. DGV for milk yield, fat content and protein content were calculated using both the actual 50K markers (DGV) and the imputed genotypes (DGV_IMP). Briefly, effects of SNP genotypes on phenotypes in the TP population were estimated using a BLUP model [24]:

y=1μ+Zg+e

where **y** is the vector of polygenic breeding values, **1** is a vector of ones, *μ* is the overall mean, **Z** is the matrix of SNP scores, **g** is the vector of SNP regression coefficients assumed identically and normally distributed with *g*_i_*~ N*(0, **I***σ*_gi_^2^) where *σ*_gi_^2^ = *σ*_a_^2^/k (*σ*_a_^2^ = additive genetic variance, k = number SNP), and **e** is the vector of random residuals. The overall mean ($\hat{\mu }$) and the vector (**ĝ**) of the marker effects estimated in the TP were used to calculate the DGV for PP as:

$$\hat{y}=\hat{\mu }+Z^{*}\hat{g}$$

where **ŷ** is the vector of estimated DGV and Z^*^ is the matrix of SNP scores in PP. For each phenotype, both DGV and DGV_IMP were obtained and correlations between DGV and DGV_IMP were calculated (r).

## Results

Results obtained by analyzing Daetwyler’s data are reported in Table 1. Values of R for both PLSR and Beagle were higher than those obtained with ChromoPhase, especially for founder bulls. Nearly equal values were obtained by PLSR and Beagle for non-founder animals whereas for founders, imputation accuracy using PLSR was more than 5% higher than with Beagle.

**Table 1:** Accuracy of genotype imputation from 3K to 50K with ChromoPhase, Beagle and PLSR algorithms for founders (F) and non-founders (NF)

	**Imputation accuracy**		
**Type**	**ChromoPhase**^**1**^	**Beagle**	**PLSR**
NF	0.925	0.926	0.929
F	0.728	0.868	0.924

^1^Values from Daetwyler et al. [6].

Table 2 contains accuracies obtained with PLSR and Beagle for imputation from 3K and 7K SNP chips to 50K based on the 2093 Holstein bulls. The average R using PLSR was 89.6% (± 1.6%) and 94.2% (± 1.0%) for imputation from 3K and 7K chips, respectively. Accuracies obtained with PLSR were 4% higher than with Beagle for both LDP. As expected, R for each chromosome was higher for imputation from 7K than for imputation from 3K. For both LDP, imputation accuracies were higher for chromosomes with a high number of SNPs. For example, R was more than 4% higher for BTA1 than for BTA28, for imputation from 3K (Table 2). Finally, R obtained by combining SNPs on BTA27, 28 and 29 was 87.4%, which was nearly equal to the average R of the three chromosomes (87,3%), indicating that no advantage was obtained by combining markers from multiple chromosomes.

**Table 2:** Number of SNPs per chromosome in the 50K, 3K and 7K SNP panels and the accuracy of imputation based on 3K and 7K panels with PLSR and Beagle

	**Number of SNP**				**Imputation accuracy (PLSR)**		**Imputation accuracy (Beagle)**	
**Chromosome**	**50K**	**3K**	**7K**		**3K**	**7K**	**3K**	**7K**
1	2814	146	320		0.916	0.953	0.876	0.919
2	2294	119	277		0.911	0.951	0.863	0.922
3	2191	107	261		0.897	0.944	0.846	0.898
4	2123	106	237		0.903	0.941	0.861	0.908
5	1812	107	233		0.912	0.948	0.872	0.912
6	2164	109	254		0.908	0.953	0.867	0.914
7	1876	95	215		0.908	0.949	0.858	0.915
8	2026	104	232		0.919	0.953	0.872	0.915
9	1708	92	214		0.904	0.949	0.851	0.909
10	1841	97	209		0.909	0.946	0.872	0.915
11	1913	91	222		0.901	0.947	0.862	0.914
12	1408	85	175		0.903	0.942	0.856	0.899
13	1486	75	166		0.910	0.949	0.860	0.911
14	1453	70	166		0.897	0.945	0.850	0.912
15	1427	74	167		0.898	0.945	0.864	0.915
16	1337	74	160		0.910	0.950	0.864	0.913
17	1367	65	156		0.888	0.936	0.842	0.900
18	1147	59	136		0.877	0.924	0.825	0.884
19	1164	56	143		0.878	0.935	0.827	0.895
20	1351	70	172		0.921	0.960	0.886	0.933
21	1170	58	134		0.881	0.934	0.832	0.899
22	1087	57	133		0.894	0.941	0.849	0.900
23	919	47	118		0.887	0.938	0.842	0.895
24	1072	54	135		0.888	0.941	0.842	0.903
25	831	41	109		0.865	0.926	0.816	0.887
26	905	45	102		0.889	0.931	0.841	0.890
27	834	41	100		0.872	0.924	0.832	0.890
28	806	46	99		0.871	0.922	0.826	0.879
29	901	47	110		0.875	0.934	0.828	0.888
**Total SNP**	43427	2237	5155	**Mean**	0.896	0.942	0.851	0.905

Imputation accuracies obtained by including the Brown Swiss and Simmental breeds, both for imputation within breed and in the multiple breed scenario, are reported in Table 3. For the 3K LDP, R was 0.88 and 0.89 for Holstein and Brown Swiss breeds, respectively, whereas R was equal to 0.83 for Simmental. Imputation accuracies from 7K to 50K were, on average, 4% higher than imputation accuracies from 3K to 50K. However, the multi-breed approach led to a considerable decrease in accuracy and to a reduction of differences in imputation accuracies between breeds, for imputation from both 3K and 7K.

**Table 3:** Average accuracy of imputation from 3K and 7K to 50K panels using single-breed and multi-breed information

	**Imputation accuracy**			
	**3K**		**7K**	
**Breed**	**Single-breed**	**Multi-breed**	**Single-breed**	**Multi-breed**
Holstein	0.882	0.806	0.914	0.837
Brown Swiss	0.893	0.827	0.921	0.858
Simmental	0.826	0.788	0.854	0.817

Accuracies of DGV predictions were moderate (Table 4), in accordance with the low number of animals in TP. However, correlations between polygenic EBV and DGV (r_EBV,DGV_) and correlations between EBV and DGV_IMP (r_EBV,DGV_IMP_) were quite similar with actual and imputed data. This result is in agreement with the relatively high correlations between DGV and DGV_IMP (r_DGV,DGV_IMP_), which were on average 0.96 across the three considered traits with the 7K LDP. However, r_DGV,DGV_IMP_ was lower when using the 3K LDP, for which r_DGV,DGV_IMP_ was on average 0.89.

**Table 4:** **Correlations of direct genetic values (DGV) with polygenic estimated breeding values (EBV) (r**_**EBV,DGV**_**) and with DGV based on imputed genotypes (DGV_IMP) (r**_**DGV,DGV_IMP**_**) for milk yield, fat content and protein content**

**Scenarios**	**Milk yield**		**Fat content**		**Protein content**	
	**r**_**EBV,DGV**_	**r**_**DGV,DGV_IMP**_	**r**_**EBV,DGV**_	**r**_**DGV,DGV_IMP**_	**r**_**EBV,DGV**_	**r**_**DGV,DGV_IMP**_
Actual data (50K)	0.58		0.45		0.44	
Imputation from 7K	0.55	0.95	0.43	0.96	0.43	0.96
Imputation from 3K	0.52	0.89	0.42	0.93	0.38	0.86

## Discussion

Results of PLSR applied to Daetwyler’s data (Table 1) showed that the method did not produce different imputation accuracies for founders and non-founders, unlike ChromoPhase and, partly, Beagle. In our analyses, we never used pedigree information. As a consequence, both founders and non-founders were handled in the same manner. However, having a parent in the reference dataset seemed to be more important when using Beagle than when using PLSR. This is probably due to the different algorithms implemented in Beagle [9] and PLSR [19,25].

PLSR imputation accuracies, from 3K and 7K LDP to the 50K panel, were higher than accuracies obtained with Beagle and ChromoPhase. These results indicate that, if no pedigree information is available, the PLSR method should be preferred over the other methods studied here when imputation is from 3K or 7K to 50K.

PLSR was further used to impute SNP genotypes both in single and multi-breed scenarios based on Holstein, Simmental and Brown Swiss data sets. No MAF threshold was applied in the editing procedure. To investigate whether differences in imputation accuracies between PLSR and the Beagle algorithms could arise with edits based on MAF, the impact of several MAF thresholds (no limit, 0.01, 0.05, 0.10) was evaluated. However, no differences in imputation accuracies were observed between the PLSR and Beagle results.

Mean R values obtained with PLSR in the single-breed scenario were 89,6% and 94,2% for the 3K and 7K LDP, respectively. It is worth mentioning that, in the present study, the ratio between the number of animals (*n* = 2179 Holstein bulls) involved in the study and the mean number of markers (*m =* 1497) on each chromosome, *R*_*n/m*_, was 1.45. Dimauro et al. [18], tested the PLSR imputation method on a simulated data set with *m* = 1000 markers on a chromosome and *n* = 5865 individuals. The resulting *R*_*n/m*_ was 5.9. In ordinary statistics and, even more, in multivariate statistics, the availability of a larger number of observations guarantees more accurate results. Thus, Dimauro et al. [18] applied the PLSR method in a more optimal dataset, obtaining an imputation accuracy of 0.86. Even if the latter study and the present research are difficult to compare, the large difference between *R*_*n/m*_ ratios suggests that PLSR also works properly with actual data. This is an important result because, if a particular technique gives good results when applied to simulated data, it is not obvious that similar performances are obtained with actual data.

PLSR is an ordinary statistical technique included in the most popular commercial and free software packages that are currently used to perform genomic data analyses, such as SAS^®^ and R. The PLSR approach could thus be easily implemented in software for genomic evaluations previously developed. Moreover, with PLSR, the computing time needed to impute SNP genotypes was, on average, around 10 times lower than with Beagle. For example, with the 7K LDP, PLSR took around 1 h to impute SNP genotypes for the first chromosome, whereas Beagle needed around 8 h. This aspect should not be underrated when an algorithm is chosen to perform imputation. In particular, PLSR could probably be used to impute SNP genotypes from the 50K chip to the denser Illumina 777K platform in a reasonable amount of time.

Imputation from 7K to 50K (R = 0.94) was more accurate than imputation from 3K to 50K (R = 0.90). This is an expected result and it is comparable to that obtained by Mulder et al. [26], who found a mean imputation accuracy of around 88% for 3K and 92% for 7K, respectively. The mean R for each chromosome (Table 2) showed that genotype imputation accuracy depends strongly on the number of SNP variables in the **X** matrix. For example, in the 3K panel, BTA1 and BTA25 have 146 and 41 SNPs, respectively, and the related values of R were 0.92 and 0.87. Dimauro et al. [18] found that imputation accuracy increases as the number of extracted latent factors in the PLSR procedure increases. The maximum number of possible latent factors is lower than or equal to the number of variables in **X**. This can explain the lower imputation accuracy for chromosomes with a lower number of markers. Moreover, the dimension of **X** cannot be artificially enlarged by using SNP from several chromosomes because it resulted in an accuracy that was equal to the mean of accuracies obtained with each chromosome. This result suggests that a chromosome can be considered as a genetically and statistically independent unit.

Results for imputation based on information from multiple breeds obtained in this study, basically confirm previous reports. Values of R using multi-breed information (Table 3) were considerably lower than R for imputation within breeds. Similarly, Hayes et al. [23] obtained no advantage or, sometimes, worse results, for imputation based on information from multiple breeds, compared to single-breed information. Also, R for Simmental was lower than R for the other breeds. Dassonneville et al. [27] also reported lower imputation accuracies in the French Blonde d’Aquitaine beef breed (around 5%) than in two dairy breeds. The lower imputation accuracy for Simmental may be partially explained by the fact that the Illumina 50K platform was not tested on the Simmental breed [28] and that the effective population size of the three breeds is very different, being higher for the Simmental than the other breeds [29-31]. Differences in the underlying structure [32] of the three populations may impact imputation accuracies. Finally, the use of a multi-breed TP also did not give better accuracies in GEBV prediction than the single-breed scenario [22,33].

The impact of the SNP genotype imputation on the accuracy of DGV was small. Correlations between DGV and DGV_IMP were, on average, 0.96 for all traits for imputation from 7K to 50K, and 0.89 for imputation from 3K to 50K. Similar results were obtained by Berry and Kearney [34], who reported an average correlation of 0.97 across 15 traits for the 3K LDP. The lowest correlations between DGV and DGV_IMP were observed for imputation from 3K to 50K for protein content (0.86) and milk yield (0.89). The correlation between DGV and DGV_IMP was approximately the same (around 0.96) for all traits, when imputation was from 7K to 50K. Weigel et al. [13] reported similar values, both for milk yield and protein content, and confirmed that DGV_IMP predictions improve if the number of SNPs on the LDP increases, both for protein content and milk yield. Therefore, the 7K chip seems to be an efficient imputation tool and the imputed genotypes could be used to correctly estimate DGV for milk yield, and fat and protein content.

## Conclusions

This study demonstrates that the PLSR imputation method can efficiently impute missing genotypes from LDP to HDP. With this method, the same good results are obtained whether animals in the PP have parents in the TP or not. Moreover, the computing time was markedly lower than with Beagle. The PLSR method was applied chromosome-wise and the results indicate that imputation accuracies are higher when the number of SNPs in the **X** matrix is high. However, combining markers from several chromosomes did not increase the accuracy of imputation, which confirms that chromosomes are independent genetic and statistical units. The 7K LDP gave good results both in terms of R and DGV prediction. Similar to the 3K LDP, the multi-breed approach applied to the 7K scenario, did not yield better results than the single-breed approach.

## Competing interests

The authors declare that they have no competing interests.

## Authors’ contributions

CD conceived the original ideas and wrote, under the supervision of NPPM and PAM, the first version of the SAS code. MC, RS and GG performed the analysis. GM contributed to the development of the ideas and algorithms. CD, MC and NPPM wrote the draft of the paper and all authors contributed in refining the manuscript. All authors read and approved the final manuscript.
